# Supplementary material for: Elemental Fingerprinting of Wild and Farmed Fish Muscle to Authenticate and Validate Production Method
Source: Foods. 2022 Oct 4;11(19):3081. doi: 10.3390/foods11193081 (PMC9562899; doi:10.3390/foods11193081)
Supplement: Supplementary file 1 [file foods-11-03081-s001.zip › foods-1930180-supplementary.pdf]

Table S1. Range of elemental concentrations of *Sparus aurata* muscle (mg/Kg wet weight) collected at the four sampling origins: Olhão - wild (OW), Olhão -aquaculture in earth ponds (OP), Olhão - sea cage aquaculture (OC), Madeira - sea cage aquaculture (MC).

	OW	OP	OC	MC	Overall
**As**	0.983-15.439	0.260-8.323	1.322-3.729	0.746-2.455	0.260-15.439
**Br**	1.309-9.830	0.997-3.641	1.099-3.865	1.099-5.968	0.997-9.830
**Ca**	55.910-1066.454	67.227-1051.629	52.028-452.183	26.034-327.886	26.034-1066.454
**Cl**	0-509.460	19.149-290.047	8.888-466.707	9.195-436.461	0-509.460
**Cr**	0-0.301	0	0	0-0.547	0-0.547
**Cu**	0.305-0.841	0.140-0.871	0.248-1.139	0.335-1.081	0.140-1.139
**Fe**	3.071-58.538	3.207-48.519	2.150-55.494	1.841-49.487	1.841-58.538
**Hg**	0-0.146	0-0.085	0-0.506	0-0.064	0-0.506
**K**	3881.52-9181.29	5134.68-9624.66	2074.29-8099.59	2558.683-7893.87	2074.29-9624.66
**Mn**	0-0.503	0-0.790	0-0.446	0-0.350	0-0.790
**Na**	2537.97-6054.56	2745.02-6012.44	3376.06-7532.96	2957.02-7946.94	2537.97-7946.94
**Ni**	0-0.067	0-0.313	0-0.475	0	0-0.475
**P**	952.46-4242.26	1543.85-4045.39	471.45-2817.22	477.58-3941.47	471.45-4242.26
**Pb**	0-2.703	0.127-2.065	0-1.113	0-0.605	0-2.703
**Pr**	0-1.745	0-2.665	0-1.656	0-1.623	0-2.665
**Rb**	1.962-4.829	2.280-4.418	1.949-7.635	2.244-9.223	1.949-9.223
**S**	0-4008.38	1417.75-3447.49	459.62-2277.36	456.30-3058.85	0-4008.38
**Sb**	0-3.843	0-3.677	0-3.688	0-4.664	0-4.664
**Se**	0.255-2.788	0.360-0.565	0.321-0.498	0.124-0.261	0.124-2.788
**Sm**	0-2.487	0-2.470	0-1.380	0-1.185	0-2.487
**Sr**	0-7.487	0.117-10.052	0-2.868	0-2.171	0-10.052
**Ti**	0-0.637	0-0.883	0-1.327	0-2.408	0-2.408
**V**	0-0.728	0-0.671	0	0-1.076	0-1.076
**Y**	0-5.116	0-5.218	0-5.105	0-4.629	0-5.218
**Zn**	7.321-18.807	7.678-15.488	7.400-18.369	6.502-13.386	6.502-18.807
